# Pharmacokinetic modeling of [^18^F]fluorodeoxyglucose (FDG) for premature infants, and newborns through 5-year-olds

**DOI:** 10.1186/s13550-016-0179-6

**Published:** 2016-03-17

**Authors:** Kitiwat Khamwan, Donika Plyku, Shannon E. O’Reilly, Alison Goodkind, Xinhua Cao, Frederic H. Fahey, S. Ted Treves, Wesley E. Bolch, George Sgouros

**Affiliations:** The Russell H. Morgan Department of Radiology and Radiological Sciences, Johns Hopkins University, School of Medicine, Baltimore, MD 21205 USA; Department of Radiology, Faculty of Medicine, Chulalongkorn University and King Chulalongkorn Memorial Hospital, Thai Red Cross Society, Bangkok, 10330 Thailand; J. Crayton Pruitt Family Department of Biomedical Engineering, University of Florida, Gainesville, FL 32611 USA; Division of Nuclear Medicine and Molecular Imaging, Boston Children’s Hospital, Harvard Medical School, Boston, MA 02115 USA; Division of Nuclear Medicine and Molecular imaging, Department of Radiology, Brigham and Women’s Hospital, Harvard Medical School, Boston, MA 02115 USA

**Keywords:** FDG, Pediatric imaging, Compartmental modeling, Pharmacokinetics

## Abstract

**Background:**

Absorbed dose estimates for pediatric patients require pharmacokinetics that are, to the extent possible, age-specific. Such age-specific pharmacokinetic data are lacking for many of the diagnostic agents typically used in pediatric imaging. We have developed a pharmacokinetic model of [^18^F]fluorodeoxyglucose (FDG) applicable to premature infants and to 0- (newborns) to 5-year-old patients, which may be used to generate model-derived time-integrated activity coefficients and absorbed dose calculations for these patients.

**Methods:**

The FDG compartmental model developed by Hays and Segall for adults was fitted to published data from infants and also to a retrospective data set collected at the Boston Children’s Hospital (BCH). The BCH data set was also used to examine the relationship between uptake of FDG in different organs and patient weight or age.

**Results:**

Substantial changes in the structure of the FDG model were required to fit the pediatric data. Fitted rate constants and fractional blood volumes were reduced relative to the adult values.

**Conclusions:**

The pharmacokinetic models developed differ substantially from adult pharmacokinetic (PK) models which can have considerable impact on the dosimetric models for pediatric patients. This approach may be used as a model for estimating dosimetry in children from other radiopharmaceuticals.

**Electronic supplementary material:**

The online version of this article (doi:10.1186/s13550-016-0179-6) contains supplementary material, which is available to authorized users.

## Background

The radiation exposure resulting from medical imaging has become a public safety concern [1–3]. Dose reduction for pediatric patients is particularly important since such patients are considered to be at increased risk for cancer owing to the enhanced radiosensitivity of their tissues and the longer time period over which stochastic radiation effects may manifest [4, 5].

Guidelines on the amount of activity to administer for pediatric nuclear medicine imaging are based on expert consensus of best practices [6, 7]. Methods based on balancing activity administration with whole-body photon fluence or diagnostic image quality to arrive at an optimal administered activity have also been examined [8–11]. Optimization efforts would benefit by the availability of pharmacokinetic data for radiopharmaceuticals commonly used in pediatric nuclear medicine imaging. An extensive set of absorbed dose estimates and corresponding pharmacokinetic data has been published by the International Commission on Radiological Protection (ICRP) for many radiopharmaceuticals [12, 13]. The tabulated calculations include absorbed and effective doses to children. The biokinetic models used in these calculations, however, are typically derived from adult data, and the applicability of these models to children has not been ascertained. There are a number of studies that provide pharmacokinetic (PK) data for fluorodeoxyglucose (FDG) in pediatric patients [14–19]. Few to none of these studies, however, include PK data for tissues other than brain and, in one case, bladder [15]. In this work, we derive an [^18^F]-FDG model for early-age pediatric patients (newborns to 5-year-olds) based on an established [^18^F]-FDG model applicable to adults [20], which is made applicable to pediatric patients by adjusting the model and fitting it to a combination of published data and retrospective data collected at Boston Children’s Hospital (BCH). The latter data set was also used to examine the relationship between FDG uptake in different organs and patient weight or age. Such relationships will be useful as input into image simulation and diagnostic image quality evaluation tasks as described previously [10].

## Methods

### Overall approach

To arrive at a pharmacokinetic FDG model applicable to pediatric patients, we started with a published FDG PK model applicable to adults [20]. Using data from the literature [21] and a data set from BCH, the model was adjusted and used to fit the combined measured and literature-derived data set. In consultation with the institutional review board (IRB), the use of already collected, anonymized, imaging data for the purposes of this study was deemed exempt from IRB review.

### Newborns to 5-year-olds FDG pharmacokinetic data

Thirty-five patients (19 males and 16 females; age range, 2 weeks to 5 years; mean age, 1 year 4 months; patient weight ± SD, 11.47 ± 4.73 kg) who underwent whole-body [^18^F]-FDG PET studies at Boston Children’s Hospital between November 2009 and March 2015 were used to extract organ PK. As quality control, annual tests, and SUV cross calibration consistency tests of PET/CT system have been performed by medical physicists regularly, the quantification of the FDG measurement in this study can be relied even over a time span of 6 years. Patients received 5.55 MBq/kg [^18^F]-FDG intravenously as a bolus. Except for one 2-week-old infant who received 20 MBq, patients weighing less than or equal to 4.7 kg received 26 MBq. The range of administered activities was 20 to 126 MBq. The diagnostic exams were primarily for cancer diagnosis and staging. Characteristic details of the patients are presented in Table 1. Patients fasted at least for 4–6 h before injection. Imaging was acquired at approximately 60–126 min after injection using the Biograph mCT PET/CT system (Siemens Medical Solutions). As the retrospective data were used, the variability in time was due to a number of factors, including the difference of suspected diagnosis and the practicalities associated with imaging for each pediatric patient. The majority of patients (31 of 35 patients) were scanned from the skull to the lower thigh. The whole-organ percent injected activity in various organs (brain, heart wall, lungs, kidneys, and liver) was obtained from the [^18^F]-FDG PET images. Region of interests (ROIs) were manually drawn to cover the entire brain if the field of view covered the entire brain (25 of 31 patients). Otherwise (heart wall, lungs, kidneys, and liver), we used the interpolation method to determine the organ masses in order to eradicate the uncertainty from indistinct boundary of PET images especially in pediatrics to get better results of quantification. When the field of view included only a portion of the brain, ROIs on 3–4 consecutive transaxial planes through the brain were used to measure the organ activity concentration (Bq/g assuming unit mass density). In this latter group, the activity concentrations were multiplied by the brain masses interpolated from the age- and weight-specific University of Florida pediatric phantom series by matching the patients with the closest height and weight phantom in the library [22]. This approach was also used to obtain whole-organ activity for the lungs, heart wall, kidneys, and liver. For organs other than the brain, the ICRP values were very similar to the University of Florida phantom series values, and the former were used for scaling. The regions selected for activity concentration did not include tumors, and we make the assumption that scaling these regions by whole-organ mass appropriately reflects normal tissue uptake of FDG. In the case of the heart wall, ROIs were delineated around the boundary of the heart wall in each patient. If the heart wall contour could not be distinctly differentiated from the heart region, the ROIs encompassed the whole heart instead. All measured data were decay-corrected to the time of injection for each patient. If this was not already done implicitly by the scanner, then we performed an explicit decay correction. The whole-organ activities were then divided by the administered activity and multiplied by 100 to obtain percent injected activity (%IA) as a function of time after injection.

**Table 1:** Patients’ characteristic data

Patient	Sex	Age (months)	Weight (kg)	Activity (MBq)	Acquisition time after injection (min)	Provisional/suspected diagnosis (reason for PET/CT examination)
1	F	11	8.3	51.8	93	Rhabdomyosarcoma^a^
2	F	16	10.4	57.3	83	Neurofibromatosis 1 (abdominal/right flank pain)
3	F	3	4.5	27.1	94	Ewing’s sarcoma^b^
4	F	4	7.3	38.4	79	Suspected pelvic carcinoma
5	F	10	5.9	31.0	69	Retroperitoneal sarcoma s/p chemotherapy (assess for tumor activity)
6	F	3	4.0	25.9	60	Infantile adenocarcinoma^b^
7	F	0.5	3.1	19.6	60	Infantile myofibromatosis^a^
8	F	16	11.6	64.0	84	Rhabdomyosarcoma^a^
9	M	6	10.0	58.8	126	Suspected left scalp Ewing’s sarcoma
10	M	9	7.7	44.4	97	Suspected pheochromocytoma^a^
11	M	8	9.6	50.0	125	Suspected malignant liver lesion^b^
12	F	36	12.4	80.3	74	Rhabdomyosarcoma s/p therapy^a^
13	F	36	12.0	69.5	86	Rhabdomyosarcoma s/p therapy^b^
14	F	36	11.6	64.0	71	Rhabdomyosarcoma s/p therapy^b^
15	F	12	9.1	51.0	66	Infantile fibrosarcoma of pelvis s/p therapy^a^
16	M	60	18.0	64.2	70	Diffuse large B cell lymphoma stage IV^a^
17	M	7	8.6	30.6	78	Hodgkin’s lymphoma s/p chemotherapy^a^
18	F	48	16.0	91.2	81	Localized Ewing’s sarcoma^a^
19	M	48	15.1	82.9	73	Neurofibromatosis 1 (tumor activity evaluation)
20	F	13	7.3	40.7	60	Suspected malignant pelvic mass^b^
21	M	24	12.4	81.2	85	High-risk neuroblastoma s/p therapy^a^
22	M	9	10.5	94.8	113	Multifocal inflammatory myofibroblastic tumor of the lungs s/p chemotherapy^a^
23	F	10	9.9	58.8	109	B cell lymphoma^a^
24	F	7	8.1	48.1	117	Anaplastic large-cell lymphoma s/p therapy^a^
25	F	11	7.0	37.4	88	Hepatoblastoma s/p chemotherapy^a^
26	F	11	10.5	57.1	106	Suspected left renal cell carcinoma^a^
27	M	23	12.6	79.1	89	Suspected rhabdomyosarcoma
28	M	24	12.7	75.6	69	Right calf alveolar rhabdomyosarcoma^a^
29	M	36	13.8	81.4	117	Stage IV neuroblastoma s/p therapy^a^
30	M	36	14.3	74.0	83	Stage IV neuroblastoma s/p therapy^a^
31	M	48	21.7	121.4	82	Rhabdomyosarcoma^a^
32	M	60	22.0	125.8	119	Spinal neurofibroma^a^
33	F	60	20.1	116.6	81	Suspected neck LN in thyroid cancer^b^
34	M	60	16.6	92.9	83	Metastatic glomus tumor^a^
35	M	60	16.8	96.9	75	Malignant rhabdoid tumor s/p therapy^a^

^a^Evaluation for staging and/or response (post therapy)

^b^Evaluation for metastasis

### FDG compartmental model for premature infants

A compartmental modeling package, SAAM II (The Epsilon Group, Charlottesville, VA), was used for model fitting [23]. We used the whole-body adult FDG pharmacokinetic compartment model developed by Hays and Segall [20] and fitted it to partial data collected from infants. The premature infant pharmacokinetic data were derived from a report published by Niven and Nahmias [21]. In brief, these authors collected two consecutive 45-min dynamic PET scans in very low birth weight infants. The first scan was over the head, and the second was over the chest region. The time-activity curves for the brain, heart wall, lungs, and kidneys were then generated.

Model fitting to the premature infant data was obtained by adjusting the adult model parameters of each compartment that directly exchanges FDG with the plasma. The exchange rate between the plasma and erythrocyte compartments was also adjusted in this initial fitting phase. These initial fits were performed using brain FDG exchange values obtained from Huang et al. [24] which consisted of gray matter and white matter with bidirectional exchange of FDG between plasma and rapidly and slowly exchanging FDG compartments. We eliminated the distinction between white and gray matter and only retained the distinction between rapidly and slowly exchanging brain compartments. The fraction of blood volume was also gradually adjusted in order to obtain the best fit of the brain compartment model.

In fitting the lungs and heart wall, we expanded the model from a single compartment sink to two compartments that exhibit bidirectional exchange of FDG with the plasma. The fraction of blood volume in each compartment was also adjusted. The urine compartment in the adult FDG model was modified to represent the kidneys, and a bidirectional exchange with the plasma was added. The bidirectional rate constants between the plasma and kidneys were also adjusted. The compartmental structure associated with the liver and other tissues was retained as described in the adult FDG model.

### FDG compartmental model for 0- to 5-year-olds

To create the pediatric (newborns to 5-year-olds) model, the FDG model of Hays and Segall was initially fitted to the pharmacokinetic data reported by Niven and Nahmias, as described above, and then to the data obtained from BCH. The compartmental structures were kept in accordance with the infant model. As the acquisition time spanned a range between 60 and 126 min after injection for 35 patients, the data were binned into 5-min intervals and the mean and a standard deviation for the data falling into each bin was calculated and used as part of the model fitting process. Human FDG biodistribution data at multiple time points are not available for pediatric patients. As a result, the data obtained from multiple patients spanning different acquisition times were fitted into the model. We adjusted the transfer rate constant parameters between the compartment gradually for the brain, lungs, heart wall, kidneys, and liver to fit the model to these data. The SAAM II software will then generate time-integrated activity curve of the model fitted to the observed data based on a nonlinear least-squares regression algorithm. The blood volume fraction in each compartment, representing the blood physically contained in an organ or tissue relative to the total-body blood volume, was also changed. The kinetic parameters associated with rapidly and slowly exchanging tissue compartments were retained as in the infant model; however, the bidirectional exchange of the FDG between the plasma and erythrocytes had to be adjusted. The differential equations and parameter definitions describing both models are provided in Additional file 1.

### Fits to organ concentration vs weight

Imaging data obtained from BCH were nominally collected at a single time point. As noted above, the actual imaging times ranged from 60- to 126-min post-injections. To examine the relationship between organ activity concentration and patient weight, we binned the imaging data to two time intervals, 60 to 81 min and 82–126 min with 15 and 20 data points in each bin, respectively. For each time interval, the following function was fitted using the MATLAB program to obtain the organ activity concentration vs whole-body mass data set:

1 $$ \%\mathrm{I}\mathrm{A}/\mathrm{g}=a\bullet {\mathrm{weight}}^b+c\bullet {\mathrm{weight}}^d $$

where *a*, *b*, *c*, *d* are the fitted parameter values. Binning the data in two different time-interval lengths would be useful for observing the different results of the percent injected activity of the FDG uptake in each organ at the early time (60–81 min) and later time (82–126) period for generating the image simulation in the future study.

## Results

**Fig. 1:**
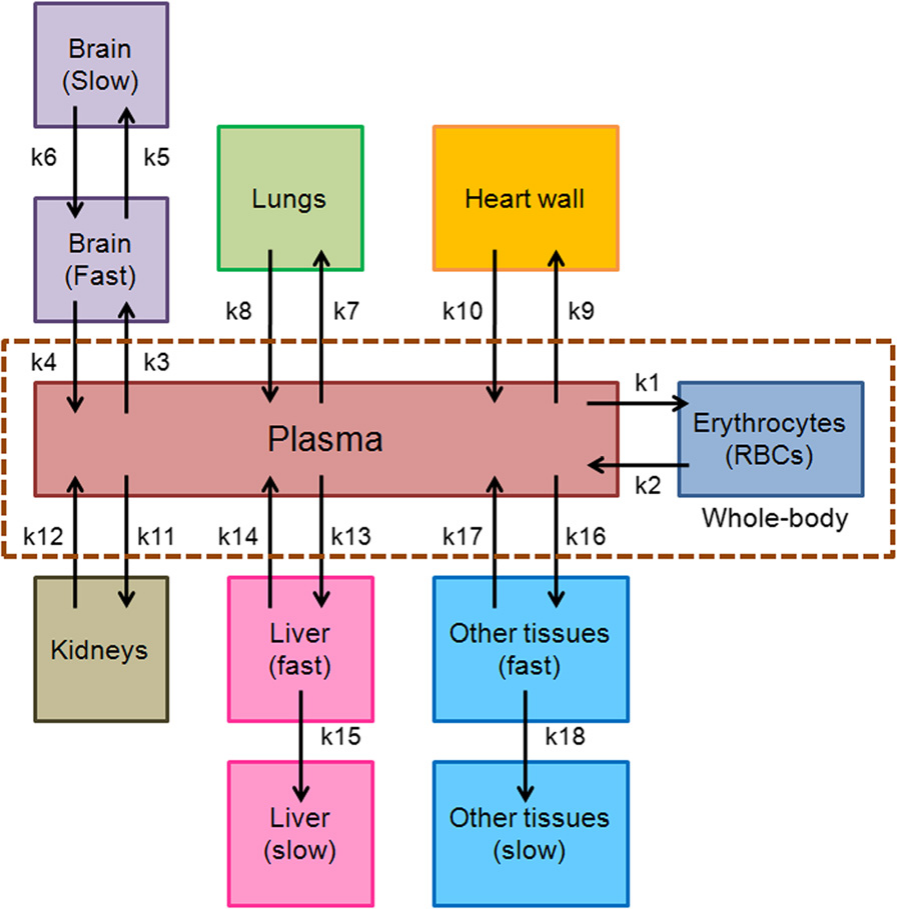
FDG compartment model used to fit the kinetic data in premature infants and newborns to 5-year-olds

**Fig. 2:**
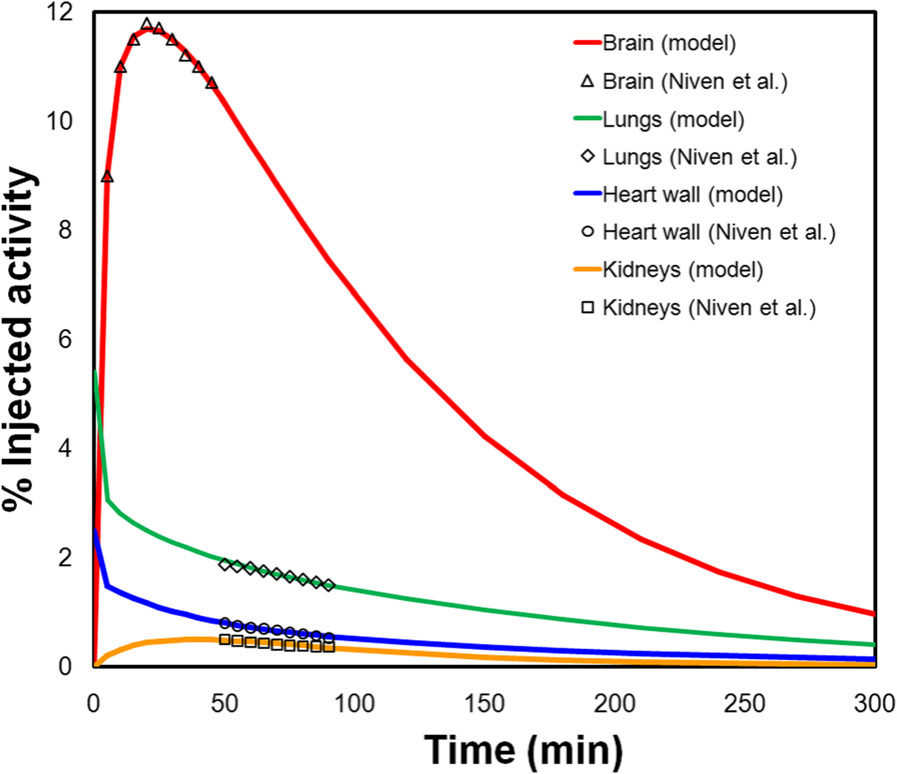
Plot of time-activity curves of the source organs that derived from the premature infant model

**Fig. 3:**
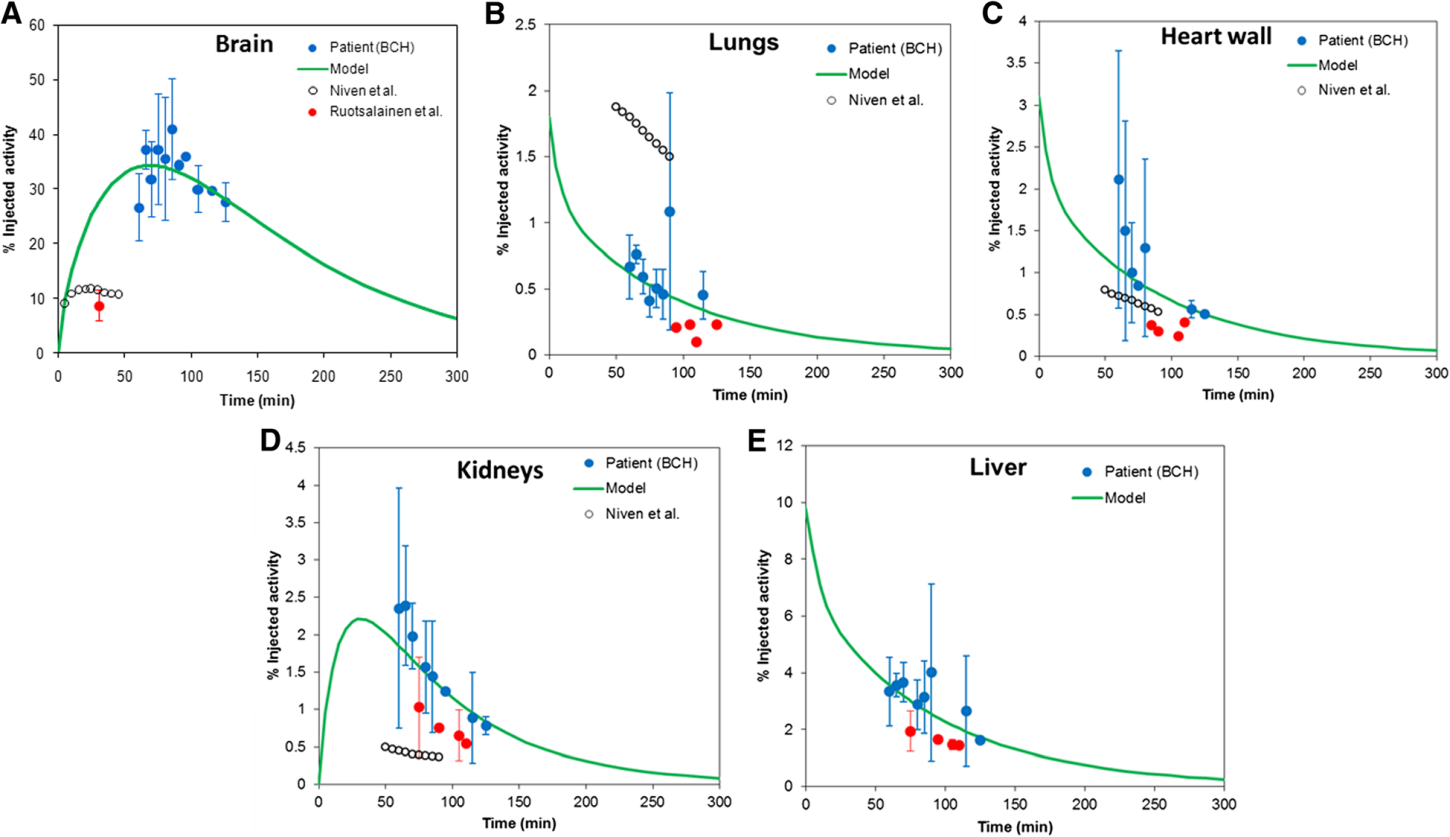
**a–e** Plot of BCH data and model-derived curves obtained from of each source organ. The *error bars* represent the standard deviation for each time point derived from the variability of %IA in multiple patients in each bin of 5-min intervals. The SD was also considered for the compartmental model fitting. In cases when literature data are available (e.g., brain, lungs, heart wall, and kidneys), these data points have been plotted to compare with the model fit to the BCH data. In the lungs, heart wall, kidneys, and liver, data points that are exclusively derived from patients <1 year old (newborns) are indicated in *red*. Other points (*blue*) are a composite of binned newborns and 1- to 5-year-olds

Figure 1 depicts the compartmental model obtained by the process described above. Figure 2 depicts the fits obtained for the premature infants. Figure 3 shows the fits obtained from the BCH data set. The error bar represents the standard deviation of %IA for each time point obtained from multiple patients. The BCH data are at the model-derived maximum FDG uptake in brain, ranging from 25 to 40 %IA. When available, data points from the literature (infants and newborns) at early time are also included in these plots for comparison (Fig. 3a). In the brain, data from Niven and Nahmias are at earlier times and fall substantially below the model fit. Likewise, the data from Ruotsalainen et al. also are well below the model fit. In the lungs (Fig. 3b), the data from Niven and Nahmias overlap in time with the BCH data and have similar clearance kinetics but are more than twofold greater, ranging from 2 to 1.5 %IA while the BCH data show lung uptake that is below 1 %IA. This is possibly because the patients in the Niven and Nahmias study suffered from lung infections. In the heart wall (Fig. 3c), the Niven and BCH data overlap with a percent uptake ranging from more than 2 %IA to less than half of a percent. The BCH data for the kidneys (Fig. 3d) range from 2.5 %IA at the earlier time interval to 0.5 %IA at the later time interval. These data may be grouped into two distinct sets, one that closely matches the PK model and another set of points with similar kinetics, but with kidney %IA that is below the first group. The latter data points (shown in red) are all obtained exclusively from newborns and are also closer to the Niven and Nahmias data points. In the liver (Fig. 3e), the model provided a good fit to the BCH data with %IA varying from slightly more than 4 to approximately 2. No literature reports of liver PK in pediatric patients could be found. As with the kidneys, data points made up exclusively from newborns were below the model fit. In Table 2, the parameter values used to fit the premature infant data of Niven and Nahmias (Fig. 2) and the retrospective BCH imaging data (Fig. 3) are compared with the original adult model parameters. The parameter set that fit the BCH data differed substantially from the adult values but was generally similar to the values obtained by fitting the premature infant data set. Table 3 lists the time-integrated activity coefficient (TIAC) obtained for each organ. These values correspond to the area under the time-activity curve of each organ. TIAC values obtained from the PK model are compared with values reported by Niven and Nahmias. In Table 4, the model-derived fractional blood volumes obtained from the model fits are compared with ICRP 106 values and with the original FDG model values. The biggest differences between ICRP 106 and the pediatric model values are for the lungs and for the brain.

**Table 2:** Parameter values fitted to the premature infants and newborns to 5-year-olds FDG model

Parameter	Premature	0- to 5-year-olds	Adults
Plasma to erythrocytes (k1)	5.82E−02	5.31E−04	4.80E+00
Erythrocytes to plasma (k2)	1.16E−01	6.88E+01	8.07E+00
Plasma to fast brain (k3)	6.12E−02	3.70E−03	1.02E−01
Fast brain to plasma (k4)	1.30E−01	1.00E−01	1.30E−01
Fast brain to slow brain (k5)	6.62E−02	1.00E+00	6.20E−02
Slow brain to fast brain (k6)	9.57E−04	9.57E−04	6.80E−03
Plasma to lungs (k7)	7.20E−04	5.00E−06	1.70E−03
Lungs to plasma (k8)	6.46E−04	6.50E−04	–
Plasma to heart wall (k9)	1.72E−04	8.00E−07	5.30E−03
Heart wall to plasma (k10)	1.16E−07	5.50E−01	–
Plasma to kidneys (k11)	7.57E−04	2.75E−03	–
Kidneys to plasma (k12)	2.26E−02	5.50E−02	–
Plasma to fast liver (k13)	1.72E−02	2.00E−02	6.80E−02
Fast liver to plasma (k14)	2.19E−02	3.00E+00	2.19E−01
Fast liver to slow liver (k15)	1.20E−06	1.50E−03	1.80E−02
Plasma to fast “other” (k16)	1.32E+00	4.20E−02	3.71E−01
Fast “other” to plasma (k17)	2.76E+00	8.90E−02	1.02E−01
Fast “other” to slow “other” (k18)	3.62E−03	9.47E−03	1.67E−02
Blood volume fraction in brain	2.20E−02	1.35E−01	2.20E−01
Blood volume fraction in lungs	9.00E−02	3.00E−02	1.50E−01
Blood volume fraction in heart	2.50E−02	3.10E−02	6.90E−02
Blood volume fraction in liver	–	9.80E−02	2.43E−01

**Table 3:** Time-integrated activity coefficient (TIAC) derived from the newborn FDG model compared with the published data

Organ	TIAC (h)			
	Hays and Segall	Niven and Nahmias	Premature	0- to 5-year-olds
Brain	2.20E−01 ± 0.09	2.82E−01 ± 0.07	2.76E−01	1.15E−00
Lungs	7.00E−02 ± 0.03	4.80E−02 ± 0.03	7.00E−02	1.90E−02
Heart wall	1.30E−01 ± 0.06	1.80E−02 ± 0.01	2.70E−02	3.20E−02
Kidneys	–	1.20E−02 ± 0.01	1.10E−02	4.60E−02
Liver	1.50E−01 ± 0.05	–	–	1.09E−01

**Table 4:** List of percent blood volume predicted by the FDG newborn model compared with values for the adult in ICRP 106 and the original FDG adult model

Organ	Fraction blood volume (%)			
	Adults (ICRP 106)	Adults (Hays and Segall)	Premature	0- to 5-year-olds
Brain	1.2	–^a^	2.2	13.5
Lungs	12.5	15	9	3
Heart wall	1.0 (same listed has coronary tissue)	6.9 (includes coronary artery)	2.5	3.1
Liver	10	24.3	–	9.8

^a^Hays and Segall paper referred to Huang et al. brain model and did not explicitly list a fractional blood volume for brain

**Fig. 4:**
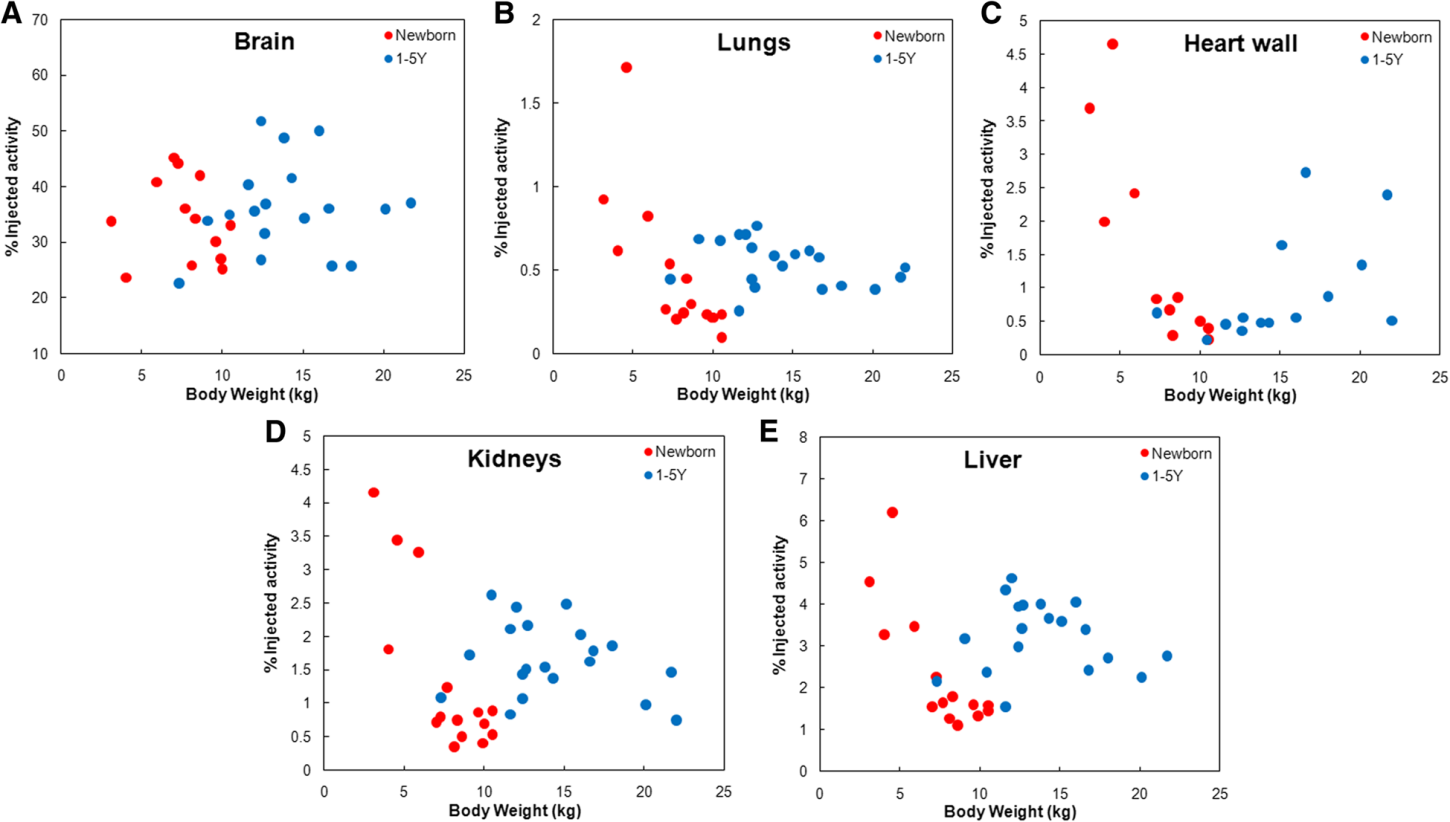
**a–e** The relationship between the patient body weight and percent injected activity in each source organ for newborns (*red*) and 1- to 5-year-olds (*blue*). Each data point corresponds to an individual patient

Figure 4 depicts the BCH data as %IA in each organ against total body weight. The plots show the expected segmentation of newborns (red) from 1- to 5-year-olds (blue) by weight. A substantial variation in FDG uptake is observed for all tissues. The greatest percent variation is in the heart wall with max (4.66 %IA) to min (0.23 %IA) ratio of approximately 20. The variation in %IA in lungs is also high with a max-to-min ratio (MMR) of 17. The variation in brain was lowest, with MMR ≈ 2. Except for brain, there was no clear trend in %IA with body weight or correspondingly a distinction between newborns and 1- to 5-year-olds. In the brain, a modest trend indicating reduced whole brain FDG uptake at lower weight and age can be discerned. In other tissues, there is a consistent pattern in which data from four low-weight newborn patients (No. 3, 5, 6, and 7 on Table 1) show a greater uptake than that seen for all of the other patients. These four patients are in large part responsible for the MMR values noted above and also for the absence of a consistent pattern in uptake vs body weight shown in Fig. 4. With these data absent, every organ shows lower FDG uptake at lower body weight.

**Fig. 5:**
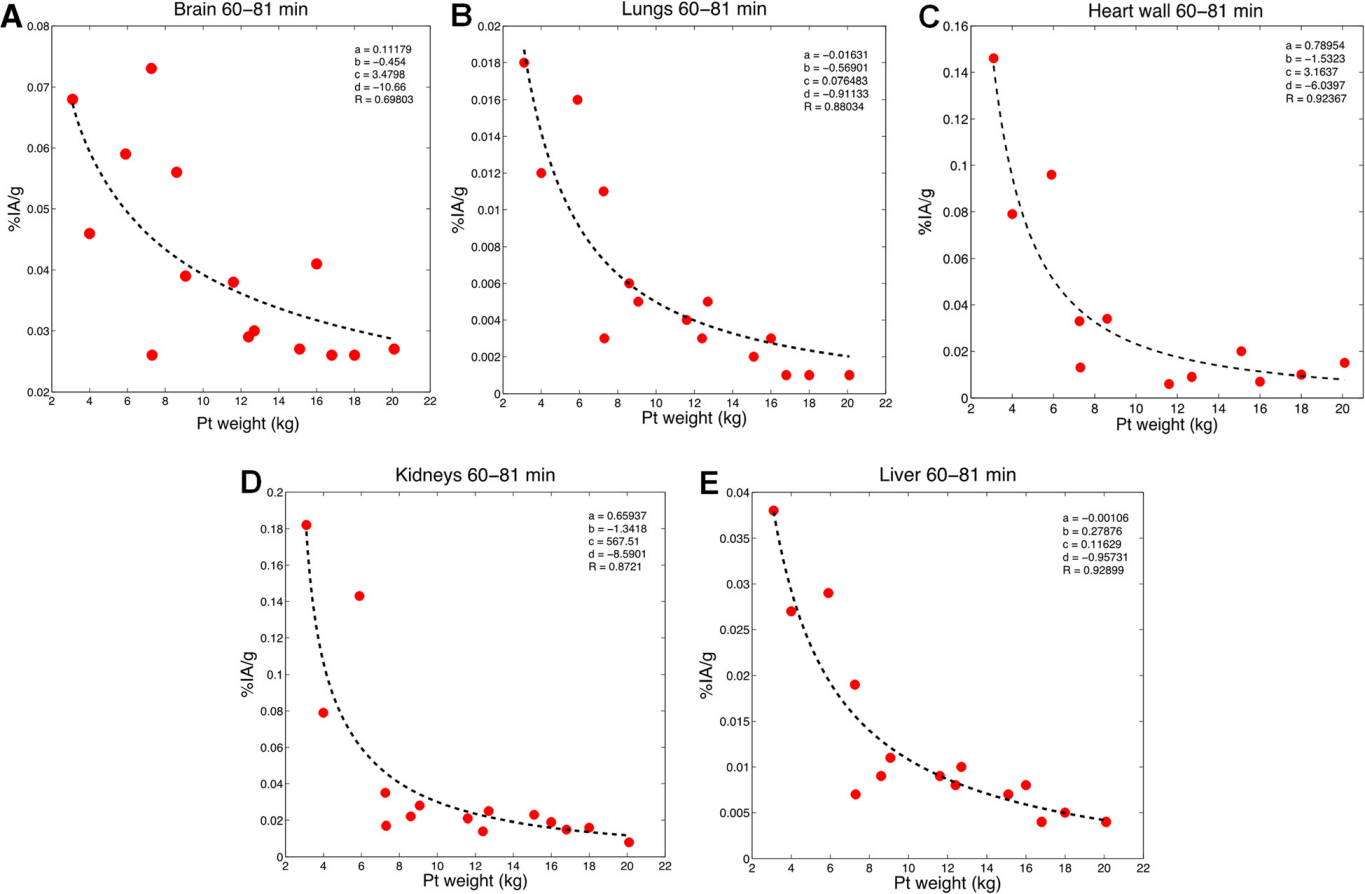
Quadratic model used to estimate %IA/g of the FDG in each organ based on a function of patient weight for 60–81 min after injection. Each data point corresponds to an individual patient

**Fig. 6:**
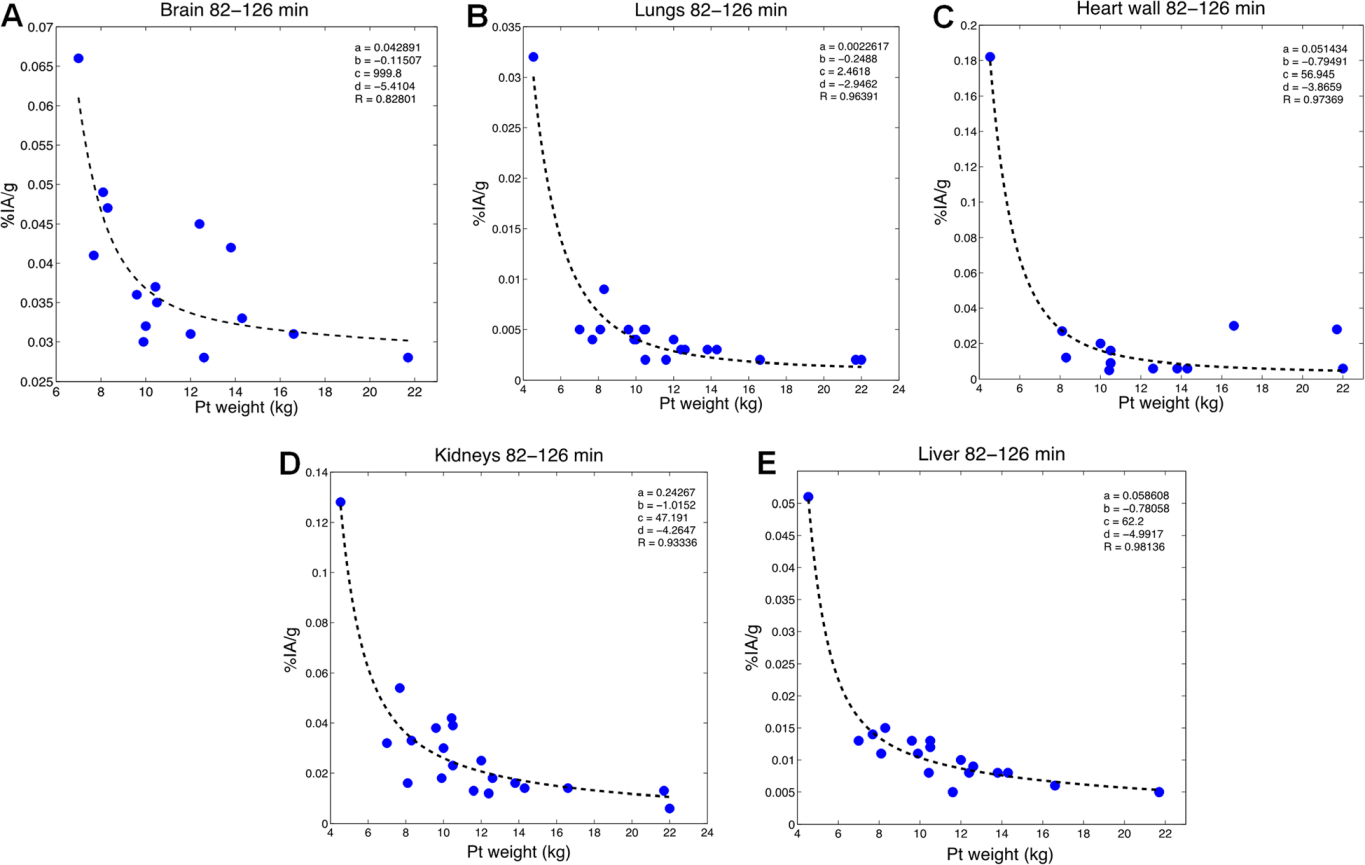
Quadratic model used to estimate %IA/g of the FDG in each organ based on a function of patient weight for 82–126 min after injection. Each data point corresponds to an individual patient

Figures 5 and 6 depict the same data set but in terms of tissue concentration rather than %IA. We examine this relationship during the earlier (60–81 min) (Fig. 5) and later (82–126 min) (Fig. 6) time period. The fit relies on the first data point for the later bin of time points, but the bin of earlier time points supports the observation at low weight. However, the data points were not weighted in the fitting process. As we do not at this time have a physiological basis for using the quadratic function, rather chose this as the best phenomenon logical fit to the observations. The latter remains useful for studies endeavoring to predict FDG concentration in different tissues of pediatric patients. During both time periods, it is clear that the concentration of FDG in each organ increases with decreasing patient weight. This relationship appears to be more robust for all organs at early time because there was a greater span in the weights available at early time (see Table 1). It is possible that this observation is a result of using a minimal administered activity for pediatric patients below a certain weight. This would increase the blood concentration with decreasing weight and is consistent with circulating blood as the main source of FDG activity in normal tissue [25]. Table 5 lists the fitted parameter values for Eq. 1 used to fit these data. The equation and parameter values can be used to estimate the concentration in different organs at the imaging time point for pediatric patients in the weight range shown. Such data are useful for image simulation studies wherein an estimate of the activity concentration in each organ as a function of patient weight is needed to generate a simulated image at the imaging time point.

**Table 5:** Fitting coefficients used to determine the %IA/g for each organ at different time point for newborn FDG model

Organ	Coefficient value			
	*a*	*b*	*c*	*d*
Brain_(60–81)_	0.1118	−0.454	3.4798	−10.66
Brain_(82–126)_	0.0429	−0.1151	999.8	−5.4104
Lungs_(60–81)_	−0.0163	−0.5690	0.0765	−0.9113
Lungs_(82–126)_	0.0023	−0.2488	2.4618	−2.9462
Heart wall_(60–81)_	0.7895	−1.5323	3.1637	−6.0397
Heart wall_(82–126)_	0.0514	−0.7949	56.945	−3.8659
Kidneys_(60–81)_	0.6594	−1.3418	567.51	−8.5901
Kidneys_(82–126)_	0.2427	−1.0152	47.191	−4.2647
Liver_(60–81)_	−0.0011	0.2788	0.1163	−0.9573
Liver_(82–126)_	0.0586	−0.7806	62.2	−4.9917

## Discussion

The goal for every pediatric molecular imaging study is to obtain the best diagnostic information employing the highest quality standards, in the shortest period of time, and with the lowest patient radiation exposure [26]. The Image Gently Campaign, an initiative of the Alliance for Radiation Safety in Pediatric Imaging, has highlighted the need to tailor diagnostic imaging procedures to children so as to reduce their radiation exposure and potential cancer risk (http://www.imagegently.org, accessed May 2015).

Almost all of the pharmacokinetic measurements available for absorbed dose and risk calculations are based on data collected from adults [13]. Using data from the literature and from retrospective measurements in different patients obtained from BCH, we have developed pharmacokinetic models for dosimetry and activity concentration as a function of body weight to be used for image simulation. Due to incomplete descriptions of acquisition parameters and possible differences in sensitivity, it is difficult to compare the data obtained from the literature with the retrospective data we collected at BCH [27]. Accordingly, we have superimposed the data from the literature, when available, with BCH data and model fits in Fig. 3 to highlight the serious need for a consistent PK data set for pediatric patients. Also, the BCH data are at a single nominal point in time but, due to the practicalities associated with imaging pediatric patients, there was a substantial variability in imaging time. This allowed us to generate kinetic data over the relative short time span defined by the BCH data set. A more comprehensive data collection effort would require an imaging protocol to image at additional time points. Finally, as shown in Table 1, each time point is derived from a single patient. Given the limitations associated with pediatric imaging, it may be difficult for a series of pediatric patients to be imaged over multiple time points; rather, data from multiple patients spanning different acquisition times will need to be assembled to establish a pharmacokinetic profile for FDG and other agents used in pediatric imaging.

A number of interesting observations may be extracted from the results presented above. We find that the %IA in the brain obtained from BCH data is greater than predicted from the adult model and also from the premature infant data. Correspondingly, the brain TIAC derived from the BCH data is approximately four times greater than the other estimates shown in Table 3. The TIAC in heart wall was about fivefold lower in the premature infants than the value for adults calculated using the Hays and Segall FDG model. Brain and lung AUC values for the fitted premature infant model were about the same as those in the adult FDG model. The TIACs obtained from fits to the BCH data (0- to 5-year-olds) differ from the premature infant fits as might be expected given the nature of the premature infant data in which these patients were being imaged due to lung infections. Accordingly, the lung TIAC in this patient population is 3.7-fold higher than that seen in the BCH data. The brain, heart wall, and kidneys are 76, 16, and 76 % higher, respectively, in the BCH data set compared to the Niven and Nahmias data set.

In Table 4, the model-derived estimates of the percent blood volume for the brain, heart wall, lungs, and liver are compared with published values. The fitted values for both the premature infants and for the BCH data set are greater for brain and heart wall relative to the values for adults reported in ICRP 106. The fractional blood volume for liver derived from the BCH data sets is the same as that reported for adults. Liver data were not reported by Niven and Nahmias. The fractional blood volume in lungs of premature infants and newborns to 5-year-old children are 40 % and fourfold lower, respectively, than the adult values reported in ICRP 106. The model-derived premature infant heart wall percent tissue blood volume is greater than in the ICRP 106 reference adult by more than a factor of two. The Hays and Segall value for heart wall is about three times greater than the value we obtained for the newborns. The newborn lung percent blood volume is lower than the value listed in ICRP 106 and in the adult FDG model. These comparisons, especially for the heart, are made difficult because of the equivocal descriptions of blood content and region described. For example, Niven and Nahmias described a region of interest over the heart for imaging-based measurements, which would presumably include both parenchymal (heart wall blood) and heart contents. The calculated TIAC, however, is ascribed to heart wall based on the assumption that little activity would be in the blood after 45 min, the time of imaging. The Hays and Segall paper provides a footnote to the listed value of percent blood volume indicating that the fractional blood volume includes blood in the coronary artery content.

Current pediatric absorbed dose estimates are performed using adult pharmacokinetic data with *S* values that account for the anatomical differences between adults and children. The divergence, in both methodology, patient population and results obtained amongst the different available sources of data for pediatric pharmacokinetic modeling of FDG, highlights the need for greater data collection of pediatric imaging agents.

## Conclusions

Model-derived extrapolation of adult pharmacokinetic data provides an initial approach to extending pediatric PK data for use in dosimetry and image simulation. Additional measurements over time are needed to further validate these pediatric FDG models.

## Additional file

Additional file 1: Pharmacokinetic model equations for premature infants and newborn through 5-year-olds. Equations from SAAM II compartment model used to derive TIAC in each source tissue (or sample) for brain, lungs, heart wall, kidneys, and liver are also provided. For each source organ or each sample, qi represents the differential equations created internally and solved by SAAM II. (DOCX 290 kb)

## References

[1] Schauer DA, Linton OW (2009). NCRP report No. 160, ionizing radiation exposure of the population of the United States, medical exposure—are we doing less with more, and is there a role for health physicists?. Health Phys.

[2] Hricak H, Brenner DJ, Adelstein SJ, Frush DP, Hall EJ, Howell RW (2011). Managing radiation use in medical imaging: a multifaceted challenge. Radiology.

[3] Mettler FAJ, Thomadsen BR, Bhargavan M, Gilley DB, Gray JE, Lipoti JA (2008). Medical radiation exposure in the US in 2006: preliminary results. Health Phys.

[4] National Research Council (2005). Health risks from exposure to low levels of ionizing radiation: BEIR VII—phase 2.

[5] ICRP (2007). Publication 103: the 2007 recommendations of the International Commission on Radiological Protection. Ann ICRP.

[6] Lassmann M, Treves ST (2014). Paediatric radiopharmaceutical administration: harmonization of the 2007 EANM paediatric dosage card (version 1.5. 2008) and the 2010 North American consensus guidelines. Eur J Nucl Med Mol Imaging.

[7] Treves ST, Lassmann M (2014). International guidelines for pediatric radiopharmaceutical administered activities. J Nucl Med.

[8] Lassmann M, Biassoni L, Monsieurs M, Franzius C (2008). The new EANM paediatric dosage card: additional notes with respect to F-18. Eur J Nucl Med Mol Imaging.

[9] Lassmann M, Biassoni L, Monsieurs M, Franzius C, Jacobs F (2007). The new EANM paediatric dosage card. Eur J Nucl Med Mol Imaging.

[10] Sgouros G, Frey EC, Bolch WE, Wayson MB, Abadia AF, Treves ST (2011). An approach for balancing diagnostic image quality with cancer risk: application to pediatric diagnostic imaging of ^99m^Tc-dimercaptosuccinic acid. J Nucl Med.

[11] Alessio AM, Sammer M, Phillips GS, Manchanda V, Mohr BC, Parisi MT (2011). Evaluation of optimal acquisition duration or injected activity for pediatric ^18^F-FDG PET/CT. J Nucl Med.

[12] ICRP (1988). Publication 53: radiation dose to patients from radiopharmaceuticals. Ann ICRP.

[13] ICRP (2008). Publication 106: radiation dose to patients from radiopharmaceuticals: addendum 3 to ICRP publication 53. Ann ICRP.

[14] Hua C, Merchant TE, Li X, Li Y, Shulkin BL (2015). Establishing age-associated normative ranges of the cerebral ^18^F-FDG uptake ratio in children. J Nucl Med.

[15] Ruotsalainen U, Suhonen-Polvi H, Eronen E, Kinnala A, Bergman J, Haaparanta M (1996). Estimated radiation dose to the newborn in FDG-PET studies. J Nucl Med.

[16] London K, Howman-Giles R (2014). Normal cerebral FDG uptake during childhood. Eur J Nucl Med Mol Imaging.

[17] Chugani HT, Phelps ME (1986). Maturational changes in cerebral function in infants determined by ^18^F-FDG positron emission tomography. Science.

[18] Chugani HT, Phelps ME, Mazziotta JC (1987). Positron emission tomography study of human brain functional development. Ann Neurol.

[19] Van Bogaert P, Wikler D, Damhaut P, Szliwowski H, Goldman S (1998). Regional changes in glucose metabolism during brain development from the age of 6 years. Neuroimage.

[20] Hays MT, Segall GM (1999). A mathematical model for the distribution of fluorodeoxyglucose in humans. J Nucl Med.

[21] Niven E, Nahmias C (2003). Absorbed dose to very low birth weight infants from ^18^F-fluorodeoxyglucose. Health Phys.

[22] Geyer AM, O’Reilly S, Lee C, Long DJ, Bolch WE (2014). The UF/NCI family of hybrid computational phantoms representing the current US population of male and female children, adolescents, and adults—application to CT dosimetry. Phys Med Biol.

[23] Barrett PHR, Bell BM, Cobelli C, Golde H, Schumitzky A, Vicini P (1998). SAAM II: simulation, analysis, and modeling software for tracer and pharmacokinetic studies. Metabolism.

[24] Huang SC, Phelps ME, Hoffman EJ, Sideris K, Selin CJ, Kuhl DE (1980). Noninvasive determination of local cerebral metabolic rate of glucose in man. Am J Physiol.

[25] Hänscheid H, Fernández M, Lassmann M (2015). The absorbed dose to blood from blood-borne activity. Phys Med Biol.

[26] Treves ST (2007). Pediatric nuclear medicine/PET.

[27] Boellaard R (2009). Standards for PET image acquisition and quantitative data analysis. J Nucl Med.

