# Supplementary material for: Pharmacokinetic modeling of [18F]fluorodeoxyglucose (FDG) for premature infants, and newborns through 5-year-olds
Source: EJNMMI Res. 2016 Mar 17;6:28. doi: 10.1186/s13550-016-0179-6 (PMC4797375; doi:10.1186/s13550-016-0179-6)
Supplement: Additional file 1: — Pharmacokinetic model equations for premature infants and newborn through 5-year-olds. Equations from SAAM II compartment model used to derive TIAC in each source tissue (or sample) for brain, lungs, heart wall, kidneys, and liver are also provided. For each source organ or each sample, qi represents the differential equations created internally and solved by SAAM II. (DOCX 290 kb) [file 13550_2016_179_MOESM1_ESM.docx]

**Supplemental Data**

*Pharmacokinetic model equations*

Abbreviations

B_f_ Fast brain

B_s_ Slow brain

E Erythrocytes

K Kidneys

L Lungs

LI_f_ Liver fast

LI_s_ Liver slow

HW Heart wall

O_f_ Fast “other”

O_s_ Slow “other”

P Plasma

1. Fast brain compartment;

$$\frac{dB_{f}}{dt}=-(k_{4}+k_{5})B_{f}+k_{6}B_{s}+k_{3}P$$

1. Slow brain compartment;

$$\frac{dB_{s}}{dt}=-k_{6}B_{s}+k_{5}B_{f}$$

1. Lungs compartment;

$$\frac{dL}{dt}=-k_{8}L+k_{7}P$$

1. Heart wall compartment;

$$\frac{dHW}{dt}=-k_{10}HW+k_{9}P$$

1. Kidneys compartment;

$$\frac{dK}{dt}=-k_{12}K+k_{11}P$$

1. Fast liver compartment;

$$\frac{dLI_{f}}{dt}=-(k_{14}+k_{15})LI_{f}+k_{13}P$$

1. Slow liver compartment;

$$\frac{dLI_{s}}{dt}=k_{15}LI_{f}$$

1. Fast “other” compartment;

$$\frac{dO_{f}}{dt}=-(k_{17}+k_{18})O_{f}+k_{16}P$$

1. Slow “other” compartment;

$$\frac{dO_{s}}{dt}=k_{18}O_{f}$$

1. Plasma compartment;

$$\begin{matrix} \frac{dP}{dt}=-(k_{1}+k_{3}+k_{7}+k_{9}+k_{11}+k_{13}+k_{16})P+k_{2}E+k_{4}B_{f}+k_{8}L+k_{10}HW \\ \text{ +}k_{12}K+k_{14}LI_{f}+k_{17}O_{f} \end{matrix}$$

1. Erythrocytes compartment;

$$\frac{dE}{dt}=-k_{2}E+k_{1}P$$

**Equations from SAAM II compartment model used to derive TIAC in each source tissue**

**Sample1 (heart wall)**

s1 = (q3 +(q1+ q2)*0.031)*exp(-log(2)*t/110)

**Sample 2 (lungs)**

s2 = (q4 + ((1-a)*q2 + a*q1)*0.03)*exp(-log(2)*t/110)

* a = 0.6

**Sample 3 (brain)**

s3 = ((q5+q6)*0.135*(a*q1+(1-a)*q2))*exp(-log(2)*t/110)

**Sample 4 (kidneys)**

s4 = (q7)*exp(-log(2)*t/110)

**Sample 5 (liver)**

s5 = ((q2 + q1)* 0.098+ q8+q9)*exp(-log(2)*t/110)

q_i_ represents the differential equations created internally and solved by SAAM II

where

q1 = plasma

q2 = erythrocytes

q3 = heart wall

q4 = lungs

q5 = fast brain

q6 = slow brain

q7 = kidneys

q8 = fast liver

q9 = slow liver
